# Aqua­[*N*-(1-naphth­yl)acetamido-κ*N*]bis­[2-(2-pyrid­yl)phenyl-κ^2^ *N*,*C* ^1^]iridium(III) ethyl­ene glycol hemisolvate

**DOI:** 10.1107/S1600536808010040

**Published:** 2008-04-30

**Authors:** Hao Fu, Yuqiang Ding, Guoqing Chen

**Affiliations:** a School of Chemical and Materials Engineering, Jiangnan University, 1800 Lihu Road, Wuxi, Jiangsu Province, People’s Republic of China; b School of Science, Jiangnan University, 1800 Lihu Road, Wuxi, Jiangsu Province, People’s Republic of China

## Abstract

In the title compound, [Ir(C_11_H_8_N)_2_(C_12_H_10_NO)(H_2_O)]·0.5C_2_H_6_O_2_, the iridium center is coordinated by two N atoms and two C atoms from two 2-(2-pyrid­yl)phenyl (ppy) ligands, one N atom from the *N*-(1-naphth­yl)acetamide ligand and one water O atom, forming a distorted octa­hedral environment. Mol­ecules are linked by inter­molecular O—H⋯O hydrogen bonds formed by the coordinated water mol­ecule and the amide O atom of the *N*-(1-naphth­yl)acetamide ligands.

## Related literature

For related literature, see: Adachi *et al.* (2000 ▶); Lamansky *et al.* (2001 ▶); Beeby *et al.* (2003 ▶); You & Park (2005 ▶); Baldo *et al.* (1998 ▶).
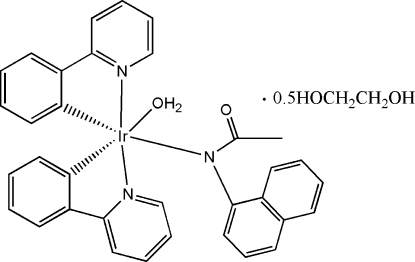


## Experimental

### 

#### Crystal data

[Ir(C_11_H_8_N)_2_(C_12_H_10_NO)(H_2_O)]·0.5C_2_H_6_O_2_
*M* *_r_* = 733.83
Triclinic, 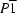

*a* = 10.097 (4) Å
*b* = 10.888 (4) Å
*c* = 14.453 (5) Å
α = 95.580 (7)°
β = 92.940 (7)°
γ = 107.423 (6)°
*V* = 1503.4 (10) Å^3^
*Z* = 2
Mo *K*α radiation
μ = 4.48 mm^−1^
*T* = 273 (2) K
0.12 × 0.10 × 0.06 mm

#### Data collection

Bruker SMART CCD area-detector diffractometer
Absorption correction: multi-scan (*SADABS*; Sheldrick, 1996 ▶) *T*_min_ = 0.615, *T*_max_ = 0.775
7940 measured reflections
5269 independent reflections
4451 reflections with *I* > 2σ(*I*)
*R*_int_ = 0.027

#### Refinement

*R*[*F* ^2^ > 2σ(*F* ^2^)] = 0.038
*wR*(*F* ^2^) = 0.093
*S* = 1.00
5269 reflections
381 parameters
H-atom parameters constrained
Δρ_max_ = 0.74 e Å^−3^
Δρ_min_ = −0.91 e Å^−3^

### 

Data collection: *APEX2* (Bruker, 2005 ▶); cell refinement: *APEX2*; data reduction: *APEX2*; program(s) used to solve structure: *SIR97* (Altomare *et al.*, 1999 ▶); program(s) used to refine structure: *SHELXL97* (Sheldrick, 2008 ▶); molecular graphics: *SHELXTL* (Sheldrick, 2008 ▶); software used to prepare material for publication: *WinGX* (Farrugia, 1999 ▶).

## Supplementary Material

Crystal structure: contains datablocks global, I. DOI: 10.1107/S1600536808010040/sg2232sup1.cif

Structure factors: contains datablocks I. DOI: 10.1107/S1600536808010040/sg2232Isup2.hkl

Additional supplementary materials:  crystallographic information; 3D view; checkCIF report

## Figures and Tables

**Table:** 

Ir1—C23	1.982 (7)
Ir1—C34	1.993 (7)
Ir1—N1	2.217 (5)
Ir1—N2	2.035 (6)
Ir1—N3	2.052 (6)
Ir1—O2	2.219 (4)

**Table:** 

C23—Ir1—C34	90.5 (2)
C23—Ir1—N2	81.7 (3)
C34—Ir1—N3	80.7 (3)
N1—Ir1—O2	86.04 (19)

**Table 2:** Hydrogen-bond geometry (Å, °)

*D*—H⋯*A*	*D*—H	H⋯*A*	*D*⋯*A*	*D*—H⋯*A*
O2—H36⋯O1^i^	0.85	1.98	2.756 (7)	150
O2—H36⋯O1^i^	0.85	1.98	2.756 (7)	150

Symmetry code: (i) 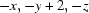.

**Fig. 1.**
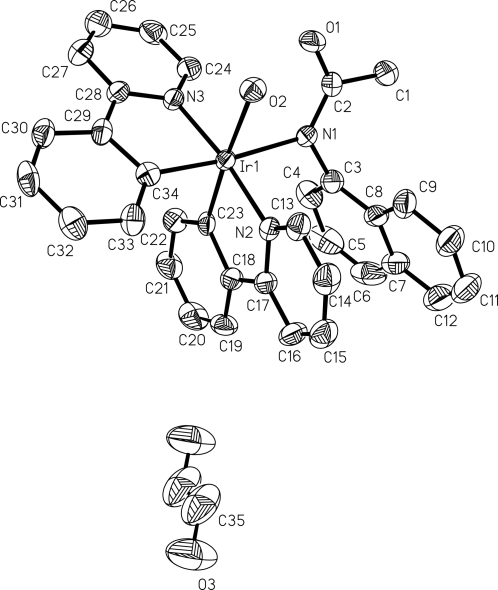
The structure of (1) showing the atomic numbering scheme and octahedral coordination of Ir(III). Non-H atoms are shown with the 30% probability displacement ellipsoids.

**Fig. 2.**
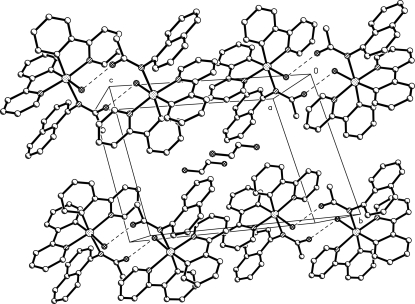
The crystal packing of (1).The intermolecluar hydrogen bonds are shown as dashed lines.

## supplementary crystallographic information

### Comment

Since the initial work by Thompson and Forrest (Baldo *et al.*, 1998), there have been considerable attention focused on designing homoleptic Ir triscyclometalates (CÑ)_3_Ir and heteroleptic Ir complexes (CÑ)_2_Ir(LX) for their application in organic light emitting diodes (OLEDs), where CÑ is a general abbreviation used hereafter for a cyclometalating ligand and LX is an ancillary ligand. (CÑ)_2_Ir(LX) complexes, containing cyclometalating ligands 2-pyridylphenyl, have already been incorporated with different kinds of ancillary ligands, such as β-diketonate, 2-picolinic acid, to exploit their potential application in OLEDs. (Adachi *et al.*, 2000; Lamansky *et al.*, 2001; Beeby *et al.*, 2003; You & Park, 2005). However, among all the ancillary lignads used in (CÑ)_2_Ir(LX) complexes, *N*-(1-naphthyl)acetamide has never been studied..

In this paper, we report the crystal structure of (CÑ)_2_Ir(LX)  with *N*-(1-naphthyl)acetamide as ancillary ligand, it is a solvated neutral mononuclear [Ir(ppy)_2_(*N*-acetyl-1-naphthylamino)(H_2_O)] (ppy=2-pyridylphenyl) complex. The Ir atom has a distorted octahedral geometry involving two ppy ligands, one *N*-(1-naphthyl)acetamide ligand and one water molecule.The average bond lengthes from two N atoms and two C atoms in two ppy ligands to iridium center are Ir—N_av_ = 2.048Å and Ir—C_av_ = 1.987Å respectively, the bond lengthes from N atom in the *N*-acetylnaphthylamine ligand and the O atom in aqua to Ir atom are Ir—N=2.217 (5)Å and Ir—O= 2.219 (4).(Table 1).

The molecules of the title complexes are linked by  O—H···O intermolecule hydrogen bonds formed by the coordinated water molecules and amido O atom of the *N*-acetyl-1-naphthylamino ligands.(Fig. 2.)

### Experimental

0.107 g [(ppy)_2_IrCl]_2_ (1 eq.) and 0.047 g (2.5 eq.) *N*-(1-naphthyl)acetamide were dissolved in dichlomethane, 0.054 g sodium methanol(10eq.) then added to the mixture to neutralize the hydrochloric acid that produced in the reaction. The reaction was stirred at room temperature for 24 h. After the reaction, the solvent was removed and the residua was washed with hot water and ether. The crude product was separated by chromatography on silica gel with dichloromethane as eluent to give a yellow solid. Single crystals suitable for X-ray diffraction were grown by slow diffusion of ethylene glycol solution.

### Refinement

All H atoms were placed in calculated positions with C—H = 0.93 (aromatic), 0.97 Å (methylene), and refined using a riding model with *U*iso(H) = 1.2*U*eq(C,*N*). Water H atoms were tentatively located in difference Fourier maps and were refined with distance restraints of O–H = 0.82 Å and H···H = 1.29 Å, each within a standard deviation of 0.01 Å; and with *U*iso(H) = 1.5 *U*eq(O).

### Crystal data

**Table:**

[Ir(C_11_H_8_N)_2_(C_12_H_10_NO)(H_2_O_1_)]·0.5C_2_H_6_O_2_	*Z* = 2
*M**_r_* = 733.83	*F*_000_ = 726
Triclinic, *P*1	*D*_x_ = 1.621 Mg m^−^^3^
*a* = 10.097 (4) Å	Mo *K*α radiation λ = 0.71073 Å
*b* = 10.888 (4) Å	Cell parameters from 2801 reflections
*c* = 14.453 (5) Å	θ = 2.3–22.3º
α = 95.580 (7)º	µ = 4.48 mm^−^^1^
β = 92.940 (7)º	*T* = 273 (2) K
γ = 107.423 (6)º	Block, green
*V* = 1503.4 (10) Å^3^	0.12 × 0.10 × 0.06 mm

### Data collection

**Table:**

Bruker SMART CCD area-detector diffractometer	5269 independent reflections
Radiation source: fine-focus sealed tube	4451 reflections with *I* > 2σ(*I*)
Monochromator: graphite	*R*_int_ = 0.027
*T* = 273(2) K	θ_max_ = 25.1º
φ and ω scans	θ_min_ = 2.0º
Absorption correction: multi-scan(SADABS; Sheldrick, 1996)	*h* = −11→12
*T*_min_ = 0.615, *T*_max_ = 0.775	*k* = −12→12
7940 measured reflections	*l* = −17→8

### Refinement

**Table:**

Refinement on *F*^2^	Secondary atom site location: difference Fourier map
Least-squares matrix: full	Hydrogen site location: inferred from neighbouring sites
*R*[*F*^2^ > 2σ(*F*^2^)] = 0.038	H-atom parameters constrained
*wR*(*F*^2^) = 0.093	*w* = 1/[σ^2^(*F*_o_^2^) + (0.05*P*)^2^ + 0.001*P*]
*S* = 1.00	(Δ/σ)_max_ = 0.004
5269 reflections	Δρ_max_ = 0.74 e Å^−^^3^
381 parameters	Δρ_min_ = −0.90 e Å^−^^3^
Primary atom site location: structure-invariant direct methods	Extinction correction: none

### Special details

**Table:**

Geometry. All e.s.d.'s (except the e.s.d. in the dihedral angle between two l.s. planes) are estimated using the full covariance matrix. The cell e.s.d.'s are taken into account individually in the estimation of e.s.d.'s in distances, angles and torsion angles; correlations between e.s.d.'s in cell parameters are only used when they are defined by crystal symmetry. An approximate (isotropic) treatment of cell e.s.d.'s is used for estimating e.s.d.'s involving l.s. planes.
Refinement. Refinement of *F*^2^ against ALL reflections. The weighted *R*-factor *wR* and goodness of fit *S* are based on *F*^2^, conventional *R*-factors *R* are based on *F*, with *F* set to zero for negative *F*^2^. The threshold expression of *F*^2^ > σ(*F*^2^) is used only for calculating *R*-factors(gt) *etc*. and is not relevant to the choice of reflections for refinement. *R*-factors based on *F*^2^ are statistically about twice as large as those based on *F*, and *R*- factors based on ALL data will be even larger.

### Fractional atomic coordinates and isotropic or equivalent isotropic displacement parameters (Å^2^)

**Table:**

	*x*	*y*	*z*	*U*_iso_*/*U*_eq_	
Ir1	0.13408 (3)	0.89887 (2)	0.175881 (18)	0.03749 (11)	
O1	0.1828 (5)	1.1138 (5)	0.0220 (4)	0.0639 (15)	
O2	−0.0027 (5)	1.0120 (4)	0.1312 (3)	0.0525 (12)	
H37	0.0550	1.0351	0.0906	0.063*	
H36	−0.0767	0.9641	0.0998	0.063*	
O3	0.9250 (19)	0.519 (2)	0.6241 (11)	0.287 (10)	
H3	0.9913	0.5173	0.6586	0.431*	
N1	0.3081 (5)	1.0563 (5)	0.1332 (4)	0.0443 (14)	
N2	0.1680 (6)	0.9878 (5)	0.3092 (4)	0.0425 (13)	
N3	0.0900 (6)	0.7881 (5)	0.0484 (4)	0.0461 (14)	
C1	0.4217 (8)	1.2363 (8)	0.0431 (6)	0.068 (2)	
H1A	0.4089	1.2499	−0.0210	0.102*	
H1B	0.5047	1.2118	0.0525	0.102*	
H1C	0.4303	1.3149	0.0826	0.102*	
C2	0.2964 (7)	1.1287 (6)	0.0672 (5)	0.0473 (17)	
C3	0.4419 (8)	1.0810 (8)	0.1841 (6)	0.059 (2)	
C4	0.5249 (8)	1.0024 (8)	0.1629 (6)	0.068 (2)	
H4	0.4975	0.9413	0.1104	0.082*	
C5	0.6484 (9)	1.0104 (12)	0.2166 (9)	0.096 (4)	
H5	0.7011	0.9557	0.2010	0.116*	
C6	0.6883 (11)	1.1041 (14)	0.2944 (9)	0.106 (5)	
H6	0.7689	1.1116	0.3316	0.127*	
C7	0.6075 (9)	1.1888 (10)	0.3179 (6)	0.073 (3)	
C8	0.4834 (8)	1.1748 (8)	0.2611 (5)	0.059 (2)	
C9	0.4042 (10)	1.2587 (8)	0.2848 (6)	0.068 (2)	
H9	0.3225	1.2530	0.2496	0.081*	
C10	0.4520 (13)	1.3486 (10)	0.3613 (7)	0.104 (4)	
H10	0.4020	1.4052	0.3782	0.125*	
C11	0.5746 (15)	1.3572 (13)	0.4147 (8)	0.115 (5)	
H11	0.6039	1.4191	0.4667	0.138*	
C12	0.6483 (12)	1.2812 (13)	0.3937 (8)	0.099 (4)	
H12	0.7295	1.2894	0.4305	0.119*	
C13	0.1079 (8)	1.0713 (7)	0.3443 (5)	0.0556 (19)	
H13	0.0478	1.0968	0.3049	0.067*	
C14	0.1302 (10)	1.1234 (8)	0.4373 (6)	0.071 (2)	
H14	0.0854	1.1822	0.4595	0.085*	
C15	0.2177 (10)	1.0875 (9)	0.4957 (6)	0.074 (3)	
H15	0.2348	1.1218	0.5582	0.089*	
C16	0.2797 (9)	1.0009 (9)	0.4610 (6)	0.069 (2)	
H16	0.3401	0.9758	0.5003	0.082*	
C17	0.2551 (7)	0.9484 (7)	0.3676 (5)	0.0512 (19)	
C18	0.3088 (7)	0.8496 (7)	0.3221 (5)	0.0519 (19)	
C19	0.4038 (8)	0.7965 (9)	0.3665 (6)	0.066 (2)	
H19	0.4368	0.8249	0.4285	0.079*	
C20	0.4466 (9)	0.7041 (9)	0.3180 (8)	0.081 (3)	
H20	0.5100	0.6702	0.3469	0.097*	
C21	0.3967 (9)	0.6599 (8)	0.2261 (7)	0.074 (3)	
H21	0.4255	0.5954	0.1940	0.089*	
C22	0.3047 (8)	0.7107 (7)	0.1820 (6)	0.0573 (19)	
H22	0.2724	0.6794	0.1202	0.069*	
C23	0.2586 (7)	0.8065 (6)	0.2264 (5)	0.0430 (16)	
C24	0.1684 (8)	0.8064 (7)	−0.0248 (5)	0.0557 (19)	
H24	0.2491	0.8768	−0.0192	0.067*	
C25	0.1343 (11)	0.7259 (8)	−0.1069 (6)	0.073 (3)	
H25	0.1924	0.7396	−0.1553	0.088*	
C26	0.0123 (12)	0.6241 (8)	−0.1167 (7)	0.085 (3)	
H26	−0.0140	0.5683	−0.1720	0.102*	
C27	−0.0684 (10)	0.6070 (8)	−0.0442 (6)	0.074 (3)	
H27	−0.1516	0.5393	−0.0508	0.089*	
C28	−0.0306 (8)	0.6881 (7)	0.0407 (5)	0.0534 (19)	
C29	−0.1027 (7)	0.6709 (6)	0.1252 (5)	0.0500 (18)	
C30	−0.2245 (8)	0.5705 (7)	0.1320 (7)	0.062 (2)	
H30	−0.2696	0.5151	0.0792	0.074*	
C31	−0.2773 (8)	0.5542 (8)	0.2165 (7)	0.072 (3)	
H31	−0.3583	0.4871	0.2212	0.087*	
C32	−0.2111 (8)	0.6368 (8)	0.2951 (7)	0.070 (2)	
H32	−0.2468	0.6240	0.3527	0.083*	
C33	−0.0919 (8)	0.7385 (7)	0.2885 (6)	0.059 (2)	
H33	−0.0501	0.7947	0.3418	0.071*	
C34	−0.0326 (6)	0.7588 (6)	0.2036 (5)	0.0426 (16)	
C35	0.9687 (19)	0.537 (2)	0.5300 (13)	0.197 (10)	
H35A	0.8860	0.5390	0.4936	0.237*	
H35B	1.0322	0.6249	0.5357	0.237*	

### Atomic displacement parameters (Å^2^)

**Table:**

	*U*^11^	*U*^22^	*U*^33^	*U*^12^	*U*^13^	*U*^23^
Ir1	0.03596 (15)	0.03611 (15)	0.03817 (16)	0.00916 (10)	−0.00124 (10)	0.00195 (10)
O1	0.057 (3)	0.066 (3)	0.067 (4)	0.013 (3)	−0.012 (3)	0.025 (3)
O2	0.048 (3)	0.057 (3)	0.057 (3)	0.024 (2)	−0.001 (2)	0.004 (2)
O3	0.33 (2)	0.33 (2)	0.175 (13)	0.053 (18)	−0.046 (15)	0.122 (15)
N1	0.039 (3)	0.044 (3)	0.046 (3)	0.009 (2)	−0.004 (3)	0.004 (3)
N2	0.042 (3)	0.038 (3)	0.043 (3)	0.007 (2)	0.004 (3)	0.000 (3)
N3	0.048 (3)	0.039 (3)	0.048 (4)	0.012 (3)	−0.007 (3)	0.001 (3)
C1	0.068 (5)	0.064 (5)	0.062 (5)	0.001 (4)	0.003 (4)	0.018 (4)
C2	0.047 (4)	0.040 (4)	0.051 (4)	0.009 (3)	0.007 (3)	0.002 (3)
C3	0.048 (4)	0.060 (5)	0.058 (5)	−0.001 (4)	0.000 (4)	0.016 (4)
C4	0.056 (5)	0.071 (6)	0.084 (6)	0.021 (4)	0.022 (5)	0.023 (5)
C5	0.048 (5)	0.131 (10)	0.125 (10)	0.031 (6)	0.018 (6)	0.069 (8)
C6	0.057 (6)	0.158 (12)	0.088 (8)	−0.007 (7)	−0.003 (6)	0.069 (8)
C7	0.047 (5)	0.094 (7)	0.059 (6)	−0.013 (5)	−0.006 (4)	0.029 (5)
C8	0.051 (4)	0.068 (5)	0.044 (5)	−0.007 (4)	0.001 (4)	0.019 (4)
C9	0.075 (6)	0.055 (5)	0.057 (5)	−0.002 (4)	0.014 (4)	0.000 (4)
C10	0.129 (9)	0.087 (7)	0.056 (6)	−0.025 (7)	0.005 (6)	−0.001 (5)
C11	0.131 (12)	0.105 (10)	0.056 (7)	−0.035 (8)	−0.007 (7)	0.000 (6)
C12	0.086 (8)	0.115 (10)	0.067 (8)	−0.009 (7)	−0.010 (6)	0.015 (7)
C13	0.052 (4)	0.053 (4)	0.055 (5)	0.007 (4)	0.008 (4)	0.000 (4)
C14	0.082 (6)	0.064 (5)	0.059 (6)	0.014 (5)	0.022 (5)	−0.006 (4)
C15	0.086 (7)	0.073 (6)	0.051 (5)	0.009 (5)	0.011 (5)	−0.007 (5)
C16	0.070 (6)	0.081 (6)	0.041 (5)	0.003 (5)	−0.006 (4)	0.002 (4)
C17	0.045 (4)	0.055 (4)	0.045 (4)	0.000 (3)	0.000 (3)	0.012 (4)
C18	0.041 (4)	0.056 (4)	0.053 (5)	0.004 (3)	−0.003 (3)	0.013 (4)
C19	0.052 (5)	0.080 (6)	0.062 (5)	0.012 (4)	−0.009 (4)	0.030 (5)
C20	0.067 (6)	0.083 (7)	0.108 (8)	0.041 (5)	−0.004 (6)	0.034 (6)
C21	0.075 (6)	0.060 (5)	0.099 (8)	0.034 (5)	0.002 (5)	0.020 (5)
C22	0.058 (5)	0.056 (5)	0.061 (5)	0.022 (4)	0.005 (4)	0.006 (4)
C23	0.036 (3)	0.033 (3)	0.054 (4)	0.001 (3)	0.001 (3)	0.005 (3)
C24	0.069 (5)	0.055 (5)	0.042 (4)	0.017 (4)	0.009 (4)	0.004 (4)
C25	0.122 (8)	0.061 (5)	0.044 (5)	0.037 (5)	0.011 (5)	0.006 (4)
C26	0.140 (10)	0.052 (5)	0.058 (6)	0.028 (6)	−0.011 (6)	−0.008 (4)
C27	0.087 (6)	0.061 (5)	0.061 (6)	0.009 (5)	−0.015 (5)	−0.005 (4)
C28	0.062 (5)	0.044 (4)	0.050 (5)	0.015 (4)	−0.013 (4)	0.000 (3)
C29	0.043 (4)	0.040 (4)	0.063 (5)	0.008 (3)	0.000 (4)	0.000 (3)
C30	0.045 (4)	0.051 (5)	0.081 (6)	0.004 (4)	−0.004 (4)	0.003 (4)
C31	0.050 (5)	0.051 (5)	0.107 (8)	−0.002 (4)	0.019 (5)	0.021 (5)
C32	0.055 (5)	0.070 (6)	0.087 (7)	0.016 (4)	0.029 (5)	0.022 (5)
C33	0.049 (4)	0.052 (4)	0.074 (6)	0.012 (4)	0.016 (4)	0.000 (4)
C34	0.031 (3)	0.049 (4)	0.054 (4)	0.022 (3)	0.006 (3)	0.007 (3)
C35	0.17 (2)	0.18 (2)	0.23 (3)	0.073 (13)	−0.10 (2)	−0.035 (17)

### Geometric parameters (Å, °)

**Table:**

Ir1—C23	1.982 (7)	C13—H13	0.9300
Ir1—C34	1.993 (7)	C14—C15	1.356 (12)
Ir1—N1	2.217 (5)	C14—H14	0.9300
Ir1—N2	2.035 (6)	C15—C16	1.351 (12)
Ir1—N3	2.052 (6)	C15—H15	0.9300
Ir1—O2	2.219 (4)	C16—C17	1.393 (10)
O1—C2	1.249 (8)	C16—H16	0.9300
O2—H37	0.8500	C17—C18	1.459 (11)
O2—H36	0.8500	C18—C19	1.417 (10)
O3—C35	1.47 (2)	C18—C23	1.432 (10)
O3—H3	0.8200	C19—C20	1.358 (12)
N1—C2	1.318 (8)	C19—H19	0.9300
N1—C3	1.443 (9)	C20—C21	1.381 (13)
N2—C13	1.313 (9)	C20—H20	0.9300
N2—C17	1.374 (9)	C21—C22	1.375 (10)
N3—C24	1.348 (9)	C21—H21	0.9300
N3—C28	1.360 (9)	C22—C23	1.382 (10)
C1—C2	1.527 (10)	C22—H22	0.9300
C1—H1A	0.9600	C24—C25	1.368 (10)
C1—H1B	0.9600	C24—H24	0.9300
C1—H1C	0.9600	C25—C26	1.378 (13)
C3—C8	1.389 (11)	C25—H25	0.9300
C3—C4	1.390 (11)	C26—C27	1.354 (13)
C4—C5	1.411 (12)	C26—H26	0.9300
C4—H4	0.9300	C27—C28	1.403 (11)
C5—C6	1.397 (16)	C27—H27	0.9300
C5—H5	0.9300	C28—C29	1.453 (11)
C6—C7	1.432 (16)	C29—C30	1.394 (10)
C6—H6	0.9300	C29—C34	1.415 (9)
C7—C12	1.368 (14)	C30—C31	1.363 (12)
C7—C8	1.423 (11)	C30—H30	0.9300
C8—C9	1.413 (12)	C31—C32	1.382 (12)
C9—C10	1.365 (12)	C31—H31	0.9300
C9—H9	0.9300	C32—C33	1.386 (10)
C10—C11	1.399 (16)	C32—H32	0.9300
C10—H10	0.9300	C33—C34	1.401 (10)
C11—C12	1.293 (16)	C33—H33	0.9300
C11—H11	0.9300	C35—C35^i^	1.426 (18)
C12—H12	0.9300	C35—H35A	0.9700
C13—C14	1.388 (11)	C35—H35B	0.9700
			
C23—Ir1—C34	90.5 (2)	C15—C14—C13	119.3 (9)
C23—Ir1—N2	81.7 (3)	C15—C14—H14	120.3
C34—Ir1—N2	93.3 (3)	C13—C14—H14	120.3
C23—Ir1—N3	94.7 (3)	C16—C15—C14	118.5 (8)
C34—Ir1—N3	80.7 (3)	C16—C15—H15	120.8
N2—Ir1—N3	172.9 (2)	C14—C15—H15	120.8
C23—Ir1—N1	93.8 (2)	C15—C16—C17	121.5 (8)
C34—Ir1—N1	174.7 (2)	C15—C16—H16	119.2
N2—Ir1—N1	90.5 (2)	C17—C16—H16	119.2
N3—Ir1—N1	95.8 (2)	N2—C17—C16	119.1 (8)
C23—Ir1—O2	175.1 (2)	N2—C17—C18	113.6 (6)
C34—Ir1—O2	89.9 (2)	C16—C17—C18	127.3 (7)
N2—Ir1—O2	93.5 (2)	C19—C18—C23	120.1 (8)
N3—Ir1—O2	90.2 (2)	C19—C18—C17	124.4 (7)
N1—Ir1—O2	86.04 (19)	C23—C18—C17	115.5 (6)
Ir1—O2—H37	85.0	C20—C19—C18	119.8 (8)
Ir1—O2—H36	112.0	C20—C19—H19	120.1
H37—O2—H36	104.4	C18—C19—H19	120.1
C35—O3—H3	109.5	C19—C20—C21	120.6 (8)
C2—N1—C3	118.9 (6)	C19—C20—H20	119.7
C2—N1—Ir1	124.8 (4)	C21—C20—H20	119.7
C3—N1—Ir1	116.3 (4)	C22—C21—C20	120.3 (9)
C13—N2—C17	118.6 (6)	C22—C21—H21	119.8
C13—N2—Ir1	125.8 (5)	C20—C21—H21	119.8
C17—N2—Ir1	115.4 (5)	C21—C22—C23	122.3 (8)
C24—N3—C28	119.5 (6)	C21—C22—H22	118.9
C24—N3—Ir1	126.1 (5)	C23—C22—H22	118.9
C28—N3—Ir1	114.3 (5)	C22—C23—C18	116.9 (6)
C2—C1—H1A	109.5	C22—C23—Ir1	129.3 (6)
C2—C1—H1B	109.5	C18—C23—Ir1	113.8 (5)
H1A—C1—H1B	109.5	N3—C24—C25	122.8 (8)
C2—C1—H1C	109.5	N3—C24—H24	118.6
H1A—C1—H1C	109.5	C25—C24—H24	118.6
H1B—C1—H1C	109.5	C24—C25—C26	118.8 (9)
O1—C2—N1	121.9 (6)	C24—C25—H25	120.6
O1—C2—C1	116.8 (6)	C26—C25—H25	120.6
N1—C2—C1	121.3 (6)	C27—C26—C25	118.6 (9)
C8—C3—C4	118.9 (8)	C27—C26—H26	120.7
C8—C3—N1	120.5 (8)	C25—C26—H26	120.7
C4—C3—N1	120.4 (7)	C26—C27—C28	122.1 (8)
C3—C4—C5	123.5 (10)	C26—C27—H27	118.9
C3—C4—H4	118.2	C28—C27—H27	118.9
C5—C4—H4	118.2	N3—C28—C27	118.1 (8)
C6—C5—C4	117.1 (11)	N3—C28—C29	115.4 (6)
C6—C5—H5	121.4	C27—C28—C29	126.3 (7)
C4—C5—H5	121.4	C30—C29—C34	121.9 (7)
C5—C6—C7	121.3 (10)	C30—C29—C28	123.7 (7)
C5—C6—H6	119.4	C34—C29—C28	114.2 (6)
C7—C6—H6	119.4	C31—C30—C29	119.7 (8)
C12—C7—C8	120.5 (11)	C31—C30—H30	120.2
C12—C7—C6	120.8 (10)	C29—C30—H30	120.2
C8—C7—C6	118.6 (10)	C30—C31—C32	120.4 (7)
C3—C8—C9	121.2 (7)	C30—C31—H31	119.8
C3—C8—C7	120.5 (9)	C32—C31—H31	119.8
C9—C8—C7	118.3 (9)	C31—C32—C33	120.2 (8)
C10—C9—C8	117.7 (10)	C31—C32—H32	119.9
C10—C9—H9	121.2	C33—C32—H32	119.9
C8—C9—H9	121.2	C32—C33—C34	121.6 (8)
C9—C10—C11	121.4 (13)	C32—C33—H33	119.2
C9—C10—H10	119.3	C34—C33—H33	119.2
C11—C10—H10	119.3	C33—C34—C29	116.2 (6)
C12—C11—C10	121.7 (12)	C33—C34—Ir1	129.1 (5)
C12—C11—H11	119.2	C29—C34—Ir1	114.7 (5)
C10—C11—H11	119.2	C35^i^—C35—O3	130 (3)
C11—C12—C7	120.5 (12)	C35^i^—C35—H35A	104.8
C11—C12—H12	119.8	O3—C35—H35A	104.8
C7—C12—H12	119.8	C35^i^—C35—H35B	104.8
N2—C13—C14	122.9 (8)	O3—C35—H35B	104.8
N2—C13—H13	118.5	H35A—C35—H35B	105.8
C14—C13—H13	118.5		

Symmetry codes: (i) −*x*+2, −*y*+1, −*z*+1.

### Hydrogen-bond geometry (Å, °)

**Table:**

*D*—H···*A*	*D*—H	H···*A*	*D*···*A*	*D*—H···*A*
O2—H36···O1^ii^	0.85	1.98	2.756 (7)	150
O2—H36···O1^ii^	0.85	1.98	2.756 (7)	150

Symmetry codes: (ii) −*x*, −*y*+2, −*z*.
